# Integrins are Mechanosensors That Modulate Human Eosinophil Activation

**DOI:** 10.3389/fimmu.2015.00525

**Published:** 2015-10-20

**Authors:** Mustafa Ahmadzai, Mike Small, Roma Sehmi, Gail Gauvreau, Luke J. Janssen

**Affiliations:** ^1^Firestone Institute for Respiratory Health, St. Joseph’s Hospital, Hamilton, ON, Canada; ^2^Department of Biomedical Sciences, McMaster University, Hamilton, ON, Canada; ^3^Department of Medicine, McMaster University, Hamilton, ON, Canada

**Keywords:** eosinophils, migration, calcium, cytoskeleton, eotaxin

## Abstract

Eosinophil migration to the lung is primarily regulated by the eosinophil-selective family of eotaxin chemokines, which mobilize intracellular calcium (Ca^2+^) and orchestrate myriad changes in cell structure and function. Eosinophil function is also known to be flow-dependent, although the molecular cognate of this mechanical response has yet to be adequately characterized. Using confocal fluorescence microscopy, we determined the effects of fluid shear stress on intracellular calcium concentration ([Ca^2+^]_i_) in human peripheral blood eosinophils by perfusing cells in a parallel-plate flow chamber. Our results indicate that fluid perfusion evokes a calcium response that leads to cell flattening, increase in cell area, shape change, and non-directional migration. None of these changes are seen in the absence of a flow stimulus, and all are blocked by chelation of intracellular Ca^2+^ using BAPTA. These changes are enhanced by stimulating the cells with eotaxin-1. The perfusion-induced calcium response (PICR) could be blocked by pre-treating cells with selective (CDP-323) and non-selective (RGD tripeptides) integrin receptor antagonists, suggesting that α_4_β_7_/α_4_β_1_ integrins mediate this response. Overall, our study provides the first pharmacological description of a molecular mechanosensor that may collaborate with the eotaxin-1 signaling program in order to control human eosinophil activation.

## Introduction

Asthma is a chronic inflammatory disease characterized by airway hyper-responsiveness, reversible bronchoconstriction, and airway remodeling concomitant with marked tissue eosinophilia (1, 2). Eosinophil migration to the peripheral tissues is mediated by the eosinophil-selective chemokine, eotaxin-1 (CCL11), which binds to the highly expressed chemokine receptor 3 (CCR3) (3, 4). The pathological role of eotaxin-1 has been well established in several animal models of airway and allergic disease, in which increased eotaxin-1 and blood/sputum eosinophil levels are associated with disease severity (5).

Overall, eosinophil infiltration of the airway tissues depends on activation of cell surface integrin receptors, which modulate the firm adhesion of eosinophils to the pulmonary vascular endothelium consequent to chemokine binding (6). Integrins are diverse transmembrane receptors that modulate cytoskeletal rearrangements during eosinophil polarization, crawling, and transmigration (6, 7). Adhesion to extracellular matrix (ECM) proteins, including fibronectin and collagen, is mediated through α_4_-integrins (8, 9). The classical paradigm of leukocyte extravasation suggests that eosinophil migration begins with cells binding chemokine molecules that are immobilized on the vascular endothelium via glycosaminoglycans (GAGs), including heparan sulfate proteoglycans (HSPGs) (10–12). Studies investigating chemokine–HSPG interactions have shown that eotaxin-1 molecules oligomerize during HSPG-binding, the corollary being that eotaxin-1 interacts relatively weakly with CCR3 (13–16). For this reason, eosinophil migration may likely depend on additional stimuli that regulate eosinophil adhesiveness. Chemokine binding is thought to induce a low-to-high affinity conformational change via an inside-out signaling cascade that culminates in extension of the integrin’s extracellular ligand-binding domain (17). Once extended, ligand-binding can elicit a reciprocal, outside-in signal transduction cascade in which secondary messengers and effector proteins are recruited in order to modify the actin cytoskeletal architecture, evoking robust changes in cell shape vital to adhesion and migration (17).

Several eosinophil functions have been linked to modulation of intracellular concentration of calcium ([Ca^2+^]_i_). Several agonists that promote adhesion and migration also elicit an elevation of [Ca^2+^]_i_, including eotaxin (18, 19). In newt eosinophils, transient Ca^2+^-spikes have been shown to be important in migration and chemotaxis (20). Another study of human eosinophils found [Ca^2+^]_i_ to be elevated concurrently with cell flattening (21); however, their experimental approach of making measurements in large populations of cells averaged out the changes and may have masked important clues regarding the causal linkages between these changes. The precise role of [Ca^2+^]_i_ in regulating eosinophil function is still poorly understood.

In the present study, we used real-time confocal fluorescence microscopy to characterize the effects of fluid shear stress on the intracellular calcium ion concentration ([Ca^2+^]_i_), cell shape, and migration in individual human eosinophils, and how this might be modulated by a chemokine (eotaxin). To the best of our knowledge, our study provides the first pharmacological description of a molecular mechanosensor in human eosinophils capable of directly regulating eosinophil activation and engaging in cross-talk with the eotaxin-1 signal transduction cascade.

## Materials and Methods

### Ethics Statement

Ethical approval was granted by the institutional ethics committee (Hamilton Integrated Research Ethics Board; HIREB) to obtain blood samples from healthy volunteers. Participants provided written informed consent to participate in this study.

### Granulocyte Isolation from Peripheral Human Blood

The granulocyte layer was isolated from whole blood as per the methods described by Sedgwick et al. (22). Briefly, approximately 30 mL of peripheral blood was collected from consenting adult male or female subjects into heparin-containing vacutainers (BD Bioscience, MD, USA). The whole blood was then diluted in equal parts by volume into McCoy’s 5A Medium and mixed thoroughly. Approximately 25 mL of the diluted blood was gently layered onto 15 mL Percoll density gradients and centrifuged (Heraeus Instruments, Osterode, Germany) at 800 × *g* for 20 min. The supernatant was removed and the bottom-most layer comprising the granulocyte fraction of cells was transferred into fresh tubes. From this point onward, all samples were kept on ice.

In order to lyse the erythrocytes, an equivalent volume of chilled NH_4_Cl (155 mM) was added and the mixture was left on ice for approximately 15 min, or until it appeared blackish-red. The mixture was then centrifuged at 300 × *g* for 10 min. The supernatant was removed and the whitish pellet was gently re-suspended in 155 mM NH_4_Cl solution and incubated on ice for approximately 15 min, as described previously. The cell suspension was centrifuged and re-suspended in NH_4_Cl solution at least twice more in this manner in order to thoroughly lyse any remaining erythrocytes. After the final lysis, the bright pink granulocyte pellet was washed and re-suspended in 5 mL of MACS buffer solution.

### Eosinophil Enrichment

The granulocyte mixture was centrifuged at 300 × *g* for 10 min and the supernatant removed. Eosinophils were purified by negative selection from the granulocyte fraction using MACS magnetically-labeled CD16-selective MicroBeads (Miltenyi Biotec, MA, USA). As per the manufacturer’s instructions, cells were co-incubated with 50 μL magnetic microbeads (per 50 × 10^6^ cells) and 50 μL MACS buffer solution for approximately 60 min in the dark and on ice (22). Cells were subsequently washed with 5 mL MACS buffer before being centrifuged at 300 × *g* for 10 min. The supernatant was removed and the pellet was re-suspended in 500 μL of MACS buffer.

The magnetically labeled cell suspension was pre-filtered using MACS pre-separation filters (Miltenyi Biotec, MA, USA) in order to remove large chunks of debris. The filtered suspension was subsequently added drop-wise to an MS separatory column (Miltenyi Biotec, MA, USA), which was placed in a MACS magnetic cell separator (Miltenyi Biotec, MA, USA). Neutrophils express high levels of the CD16 molecules on their surface. As a result, neutrophils are readily extracted from the mixture as the suspension elutes through the separatory column in the presence of a magnetic field (22). The eluate is highly enriched in eosinophils and is collected and subsequently re-suspended in RPMI 1640 Media for use in fluorimetric studies. Blood samples were not differentiated with regard to the donor’s disease status. For most subjects, the final eosinophil count was found to be 1–5 × 10^6^ cells. Cell viability was assessed using a Trypan blue stain, which revealed >90% cell viability. Previous histological examination has shown these to typically comprise 90–95% eosinophils (the remainder being primarily neutrophils and basophils). Basophils also express CCR3, but constitute only a small proportion of the total granulocytes isolated by this method; neutrophils do not express CCR3 and therefore do not respond to eotaxin-1.

### Perfusion Chamber Design

A perfusion/recording chamber was constructed by applying two parallel beads of silicon approximately 1 cm apart onto a large glass slide (45 mm × 50 mm) and gently compressing another smaller glass slide (22 mm × 40 mm) on top (see Figure S1 in Supplementary Material). The vertical height of this chamber was approximately 200–300 μm. Eosinophils were applied to the bottom of this chamber before the upper glass slide was lowered into place, then given 20–30 min to settle and adhere to the bottom of the chamber before commencing recordings. The bottom glass slide was either uncoated or coated with either rat tail collagen Type I (25 μg/cm^2^) or with bovine fibronectin (2–4 μg/cm^2^), as indicated. Perfusate was introduced at one end of this recording chamber at a rate of 2–3 mL/min, and removed by suction at the other end. Media and drugs were introduced using a gravity-controlled fluid reservoir, the height of which could be adjusted to control the rate at which drugs/fluids were applied. This “sandwich preparation” provided a convenient set-up for conducting flow experiments as it resembles the parallel-plate flow chambers used by other groups to study the physiological effects of fluid shear stress on other cell populations (23). Since the dimensions of this recording chamber and the rate of perfusion might vary from day to day, it was not possible to precisely quantify the shear stress on the cells; however, both parameters were kept constant during any given experiment, and we report here only the changes in cellular responses observed within each experiment. In one follow-up experiment done to quantify the latency between onset of flow and the subsequent calcium response, we controlled the rate of inflow using a peristaltic pump.

Experiments were conducted at room temperature and typically lasted an average of approximately 45–60 min.

### Confocal Fluorescence Microscopy

Approximately 1 × 10^6^ cells were loaded with the calcium-binding dye, Fluo-3 AM (3.5 μM in DMSO with 0.01% pluronic acid) and incubated in the dark for 60 min at room temperature. Cells were viewed using an inverted Nikon Eclipse TE2000-4 microscope (Mississauga, ON, Canada) with a 20× objective. Intracellular calcium recordings were obtained using a custom-built apparatus previously described by Mukherjee et al. (24). During recordings, eosinophils were scanned with a 20 mW photodiode laser (Coherent Technologies, CA, USA) at 488 nm in the *X*- and *Y*-planes using two mirrors oscillating at 8 and 30 Hz, respectively. The fluorescence light emitted at 510 nm was measured by a photomultiplier. *Video Savant* v4.0 software (IO Industries, London, ON, Canada) was used to digitize the signal (8-bit resolution) and produce tiff images (480 × 400 pixels) at a rate of 30 Hz. Videos were created by recording filtered frames (obtained by averaging eight consecutive raw images). Unless otherwise noted, all recordings were obtained at a frame rate of 2 Hz.

### Signal Processing and Statistical Analysis

Eosinophils are highly mobile cells that rapidly change their size and position. This posed a particular challenge for precisely quantifying fluorescence in eosinophils that moved around in the field of view. To address this issue, we used the *Particle Analysis* plug-in of the open platform software, *ImageJ* v1.6r (National Institutes of Health, Bethesda, MD, USA), in order to simultaneously track the spatial location and the fluorescence intensity of a given cell at each point in time. Images were thresholded and the built-in particle analysis module of *ImageJ* was then used to generate regions of interest (ROIs) from the binary image of each cell. Using these ROIs, we measured the mean fluorescence in a given cell at each frame of the recording. Fluorescence intensity was plotted against time to produce [Ca^2+^]-tracings. We quantified changes in fluorescence before versus after a given intervention (e.g., introduction of eotaxin, or onset of perfusion) by taking the ratio of the area under the curve (AUC) in the presence of a drug (AUC_D_) over the AUC in the absence of that drug (AUC_ND_):

(1) $${\text{AUC}}_{\text{f}}\;\text{Ratio}=\frac{{\text{AUC}}_{\text{D}}}{{\text{AUC}}_{\text{ND}}}$$

The time intervals over which AUC_D_ and AUC_ND_ were measured were identical. The AUC ratio provides a succinct description of whether a response increased (AUC > 1), decreased (AUC < 1) or was unchanged (AUC = 1).

Thus, our analysis technique allowed us to measure a number of qualitative parameters, including [Ca^2+^]_i_, cell area, shape, position, and speed of movement. In some cases, however, cells could not be automatically tracked using the *ImageJ* plug-in, and were instead manually tracked using the open source *Manual Tracking* plug-in available at http://rsb.info.nih.gov/ij/plugins/track/track.html (Institut Curie, Orsay, France). In these instances, the cell’s centroid position was manually determined for each cell at frame of a recording.

GraphPad Prism 5 (GraphPad Software Inc., La Jolla, CA, USA) and SigmaPlot 12.5 (Systat Software Inc., San Jose, CA, USA) were used to conduct all statistical analyses and to generate all figures. Where applicable, data are expressed as mean values ± SEM. Multiple cells (approximately 5–10) were analyzed from a given subject, and the responses from many different subjects were pooled in order to generate this mean value. For our purposes, sample sizes refer to the number of unique subjects in which each experimental condition was replicated.

Statistical analysis involved one-way analysis of variance (ANOVA), followed by Tukey’s *post hoc* test for comparing the difference of means. In all cases, *p* < 0.05 was considered to be significant.

### Chemicals and Reagents

Percoll was obtained from VWR (Mississauga, ON, Canada) and density gradients were prepared in Dulbecco’s phosphate buffered saline (DPBS) and HBSS at a concentration of 65% (v/v). Recombinant human eotaxin-1 (CCL11) was obtained from Reprokine (Tel Aviv, Israel). NH_4_Cl lysis buffer used during granulocyte isolation contained (in mM): 155 NH_4_Cl, 10 KCO_3_ and 0.1 ethylenediaminetetraacetic acid (EDTA), pH 7.2. All reagents were dissolved in RPMI 1640 Media, which sometimes contained 10% FBS and penicillin/streptomycin (1000 IU) Fisher Scientific, Walkersville, MD, USA), or in (Ca^2+^-free) HBSS (Gibco, Grand Island, NY, USA), pH 7.2–7.4. DPBS, McCoy’s 5A Medium and fluo-3 AM were obtained from Invitrogen (Burlington, ON, Canada). CDP-323 (Zaurategrast) was obtained from Adooq Bioscience (Irvine, CA, USA). Recombinant Arg-Gly-Asp (RGD) tripeptide, rat tail collagen (Type I), and bovine fibronectin were obtained from Sigma Aldrich (Oakville, ON, Canada). MACS buffer used during eosinophil enrichment was prepared in 1× DPBS and contained the following (in millimoles): 2 EDTA and 0.5% BSA (w/v).

## Results

### Effects of Fluid Shear Stress on Eosinophil Activation

Prior to experimentation, human eosinophils were spherical, with a smooth surface, and were fluorimetrically dark with generally no spontaneous transient fluctuations in fluorescence (see Video S1 in Supplementary Material).

Upon onset of superfusion, the majority of the cells within the field of view remained adherent, and thus could be monitored continuously throughout the duration of the experiment. A smaller fraction, however, were swept away by the flow: this was much more the case for cells studied on bare glass than on fibronectin-coated glass. We analyzed these two sets of cells separately.

**Figure 1:**
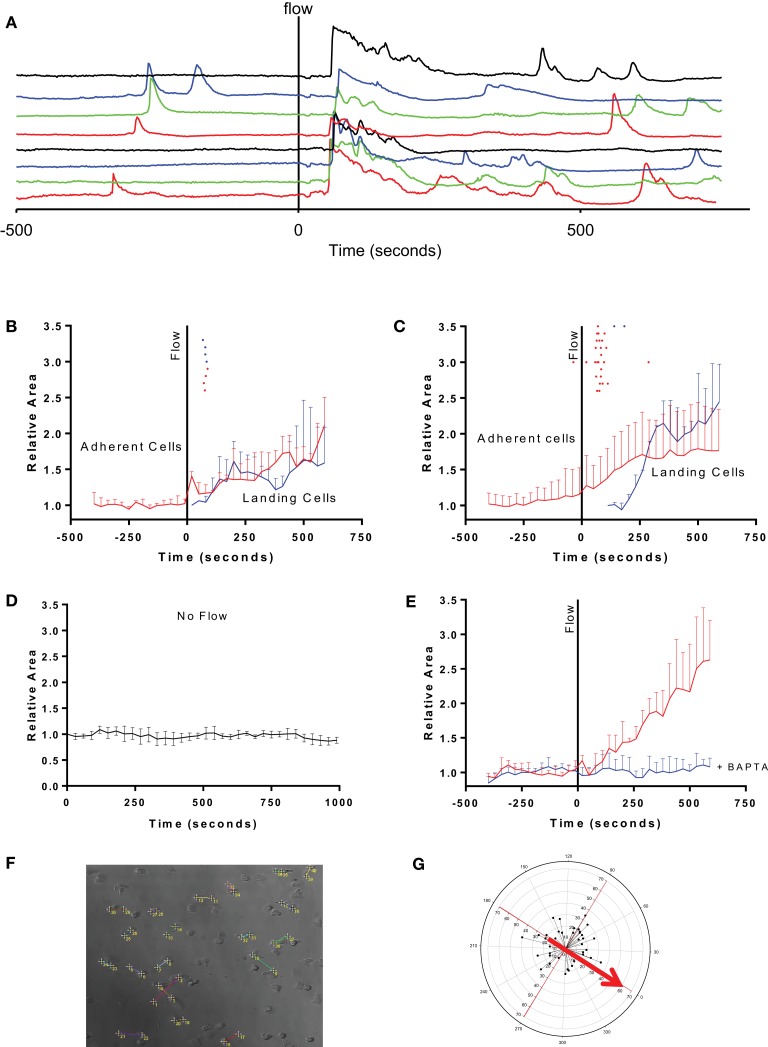
**Fluid perfusion elevates [Ca2**+**]i and induces shape change and migration in adherent human eosinophils**. **(A)** Fluorimetric tracings showing the [Ca^2+^]-elevation in response to onset of perfusion in eight different eosinophils adhered to fibronectin before and during onset of flow (indicated by vertical solid line); tracings are all displayed off-set to allow each to be easily distinguished. **(B)** Mean cell area in one preparation of eosinophils that were adherent to bare glass during onset of flow (red tracing) or that were swept into the field of view and became adherent (blue tracing). Dots above the tracings indicate the moment in time at which the PICR in each cell reached a peak. **(C)** Same analysis as in B of four other videos (29 cells) studied on fibronectin-coated glass. **(D)** Mean cell area in eosinophils adhered to fibronectin and monitored under no-flow conditions (five cells). **(E)** Mean cell area in eosinophils adhered to fibronectin after pre-loading with BAPTA-AM for 30 min prior to recording the response to flow (10 cells; blue tracing) or not loaded with BAPTA-AM (red tracings). **(F)** Representative figure showing net eosinophil displacement induced by fluid perfusion. Eosinophil displacement consequent to the PICR was assessed by determining the change in cell position over the course of the recording. The cell’s initial starting position is denoted by the lower number while the final position is indicated by the higher number. By the end of the recording, cells migrate in a direction unrelated to the direction of bulk fluid flow. **(G)** Radial plot of eosinophil displacement. The starting positions of all cells taken from at least three separate subjects were superimposed at the origin. Connecting lines adjoin the initial and final positions of all eosinophils (from at least three separate subjects) which were stimulated with RPMI perfusion. The starting location is superimposed at the origin. Red arrow denotes the direction in which fluid perfusion was applied. Circular axis: angle (in degrees) relative to direction of fluid perfusion. *X*- and *Y*-axes: distance in micrometers.

Among the cells that remained adherent throughout the recording, there was a delayed but widespread activation. That is, the majority exhibited a marked increase in fluorescence several-fold above baseline (Figure 1A), which then resolved fully back to baseline within 2 or 3 min. The delay between the onset of flow and appearance of this perfusion-induced calcium response (PICR) was flow rate-dependent: it was 267 ± 31 s at a flow rate of 0.07 mL/min (11 cells), but decreased to 110 s (1 cell) and 88 ± 40 s (10 cells) at flow rates of 0.13 and 0.20 mL/min, respectively. This PICR was often followed by one or more other transient elevations in fluorescence.

The initial PICR was also accompanied by a morphological change. In particular, the cells became less spherical, flattened out, and extruded numerous pseudopodia of varying sizes (see Video S1 in Supplementary Material). Figures 1B,C summarize the increase in cell area observed in continuously adherent cells (red traces) in cells studied on glass and fibronectin, respectively. These also mark the peaks of the PICR for each cell (red ticks). The morphological changes were much more robust in the cells studied on fibronectin (Figure 1C) compared to those studied on bare glass (Figure 1B).

The flow stimulus seems to be essential for triggering these morphological changes, since cells monitored for the same period of time under no-flow conditions did not exhibit this increase in cell area (Figure 1B). These cells also did not exhibit any appreciable changes in [Ca^2+^] (not shown).

The change in [Ca^2+^] also seems to be essential for the subsequent change in cell area, since pre-loading the cells with the Ca^2+^-chelator BAPTA fully inhibited the flow-induced change in cell area (Figure 1D).

Cell flattening and pseudopod extension were, in turn, ­followed by migration in random directions across the field of view (Figures 1E–G).

**Figure 2:**
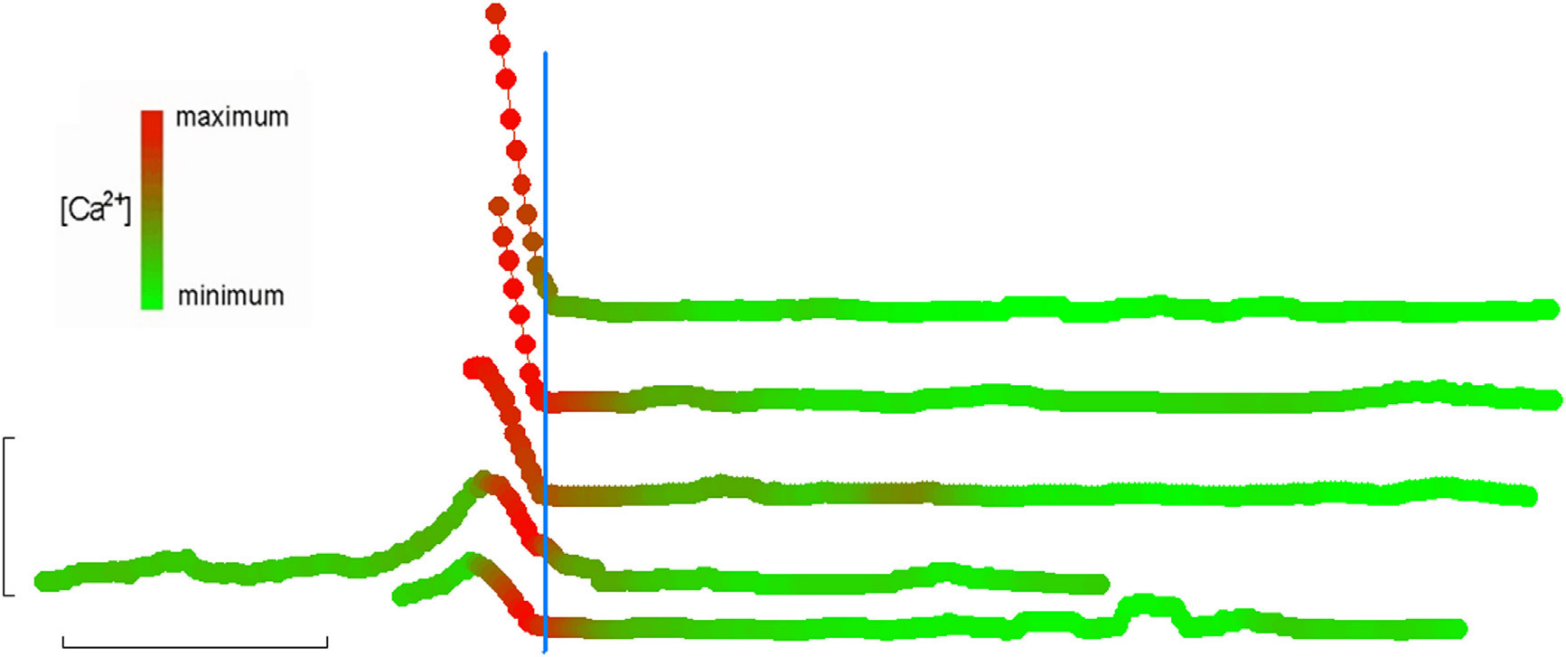
**[Ca^2+^]_i_ elevation immediately precedes adherence**. Recordings of eosinophils on fibronectin (taken from three separate subjects) that washed into the field of view immediately following onset of perfusion. Linear displacement along the direction of the flowing perfusate was determined on a frame-by-frame basis and plotted here in the vertical dimension (each tracing is also off-set in the vertical direction to allow each to be easily distinguished). Each symbol in the tracings represents an individual frame within the overall video recording. Fluorescence was also determined for each frame and is simultaneously indicated by the color of the symbol at any given frame number; for each cell, maximal and minimal fluorescence intensities were defined as 100 and 0%, respectively, and all other intensities scaled accordingly and mapped to a lookup table ranging from fully red to fully green, respectively. All such tracings were then synchronized by defining *t* = 0 as the moment when displacement first reaches 0 (indicating full adhesion). In this way, it can be seen that full adhesion is always preceded by a few seconds by a Ca^2+^-flash. Horizontal bar: 2 min. Vertical bar: 10 μm.

The second group of eosinophils that we analyzed comprised the much smaller number of cells that were swept along upon onset of flow. Although we were unable to make full-length recordings in the non-adherent cells that were swept out of the field of view (i.e., “down-stream”), we noticed other cells that had originated “up-stream” of the field of view being swept into the viewing area and becoming suddenly adherent to the glass bottom, as indicated by a relatively abrupt cessation of forward movement relative to the other debris and cells in the perfusate. We analyzed the fluorimetric changes in those cells which became adherent and stopped within the field of view, and found that all of them exhibited markedly elevated brightness which fell down to baseline levels concurrent with the cessation of their forward movement (Figure 2). In other words, it appeared that a transient PICR had occurred in all these cells as well, and resulted in immediately increased adherence to the glass substrate. In these cells too, the PICR was accompanied by the same morphological changes and migration described above for the adherent cells: Figures 1B,C also summarize the mean changes in cell area in these cells (blue traces) as well as the timing of the peaks of the PICR (blue ticks).

### Effects of Substrate Composition on Flow-Induced Responses

To examine the flow-induced responses in more detail, we compared responses in cells adhered to glass slides coated with either collagen (25 μg/cm^2^) or fibronectin (2 μg/cm^2^), which others have shown to increase adhesion and modulate function [Konya (8) 70/id; Yoshikawa (9) 131/id]. The PICR observed using these two substrates was not significantly different [1.34 ± 0.06 (*n* = 5) and 1.43 ± 0.07 (*n* = 6), respectively].

**Figure 3:**
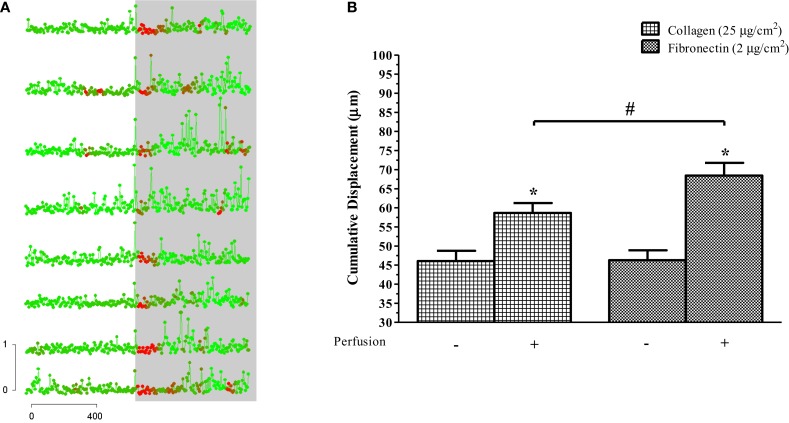
**Perfusion-stimulus doubles migration of human eosinophils**. **(A)** Displacement, measured at 8-s intervals, is plotted here against time for cells that were already adhered to the glass slide. Color of each datum point indicates the relative level of fluorescence, and the gray background indicates onset of perfusion. Horizontal bar: time in seconds. Vertical bar: distance traveled in micrometers. Points represent concatenated measurements taken at 8-s intervals and color changes reflect [Ca^2+^]_i_. Cells were taken from the same preparation but are representative of the responses seen in all flow experiments. **(B)** Mean (± SEM) cumulative displacement for eosinophils plus versus minus perfusion while on collagen- or fibronectin-coated glass slides, as indicated. *n* = 3 for each condition, where at least 5–10 cells were analyzed per sample. *p < 0.05 relative to perfusion control; ^#^*p* < 0.05 between-group treatments.

We quantified flow-induced motility as an increase in the instantaneous distance traveled over the span of the recording prior to and following onset of perfusion (Figures 3A,B). On a collagen-coated surface, eosinophils migrated a cumulative distance of 58.7 ± 2.59 and 46.1 ± 2.69 μm in the presence and absence of fluid perfusion over separate 10 min intervals, respectively (*p* < 0.05). On a fibronectin-coated surface, eosinophils migrated a cumulative distance of 68.4 ± 3.33 and 46.3 ± 2.59 μm in the presence and absence of fluid perfusion over separate 10 min intervals (*p* < 0.05). There was no significant difference between these measures of cumulative migration upon collagen versus fibronectin in the absence of flow; the marked increase in migration upon stimulation by flow, on the other hand, was significantly greater when cells were adhered to fibronectin as opposed to collagen (*p* < 0.05).

### Effects of Integrin Receptor Antagonists on the PICR

We proceeded to identify the molecular cognate of the PICR in eosinophils using a series of increasingly selective pharmacological agents. Previous studies have implicated the integrin receptor family of adhesion molecules as force transducers in various cell types, including vascular endothelial cells, neutrophils, and lymphocytes (25–27). Given that the flow-induced response in this study was largely fibronectin dependent, and that fibronectin binds primarily with β1 integrin receptor subtypes, we speculated that the latter mediate this shear-sensitivity in eosinophils. The α_4_β_7_ and α_4_β_1_ integrins overlap considerably in their ligand-binding capabilities and recognize the Arg–Gly–Asp (RGD) tripeptide motif that is expressed broadly in vascular cell adhesion molecules and/or ECM proteins (28–31). We therefore pre-treated eosinophils with soluble RGD before stimulating them with fluid shear stress.

**Figure 4:**
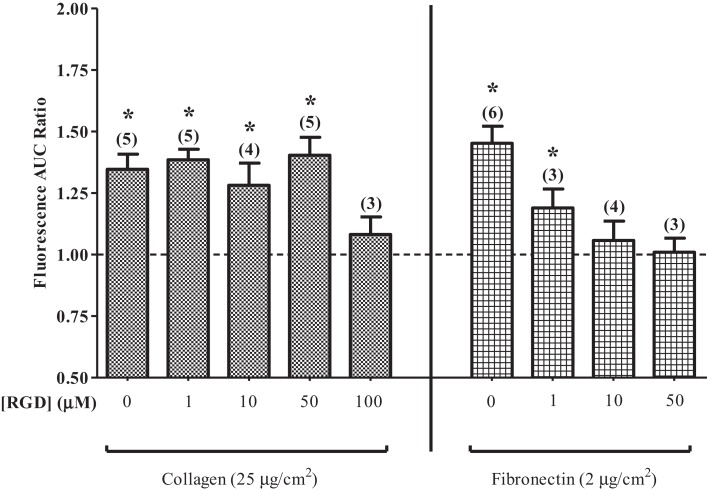
**Arg–Gly–Asp (RGD) suppresses the PICR**. PICR in cells on collagen- or fibronectin-coated glass slides in the presence or absence of various concentrations of RGD tripeptide, as indicated. Symbols indicate significant difference from unity (*p* < 0.05). *n* indicated in parentheses.

Eosinophils that were layered on fibronectin displayed a concentration-dependent decrease in the PICR following pre-treatment with RGD for 20–25 min (Figure 4). RGD failed to block the PICR in eosinophils that were layered on collagen, however, indicating that the PICR is likely mediated by a specific subset of integrin receptors that preferentially interacts with fibronectin. These findings are consistent with early functional studies in which RGD blocked keratinocyte migration on fibronectin, but failed to evoke a similar effect in the presence of collagen (32). The RGD binding site expressed in fibronectin has since been shown to compete with vascular cell adhesion molecule 1 (VCAM-1) for binding to integrins, suggesting that VCAM-1 and fibronectin interactions involve overlapping or identical binding sites (33, 34). Fibronectin best approximates the physiological milieu encountered by the eosinophil during extravasation and was therefore used in all subsequent experiments.

**Figure 5:**
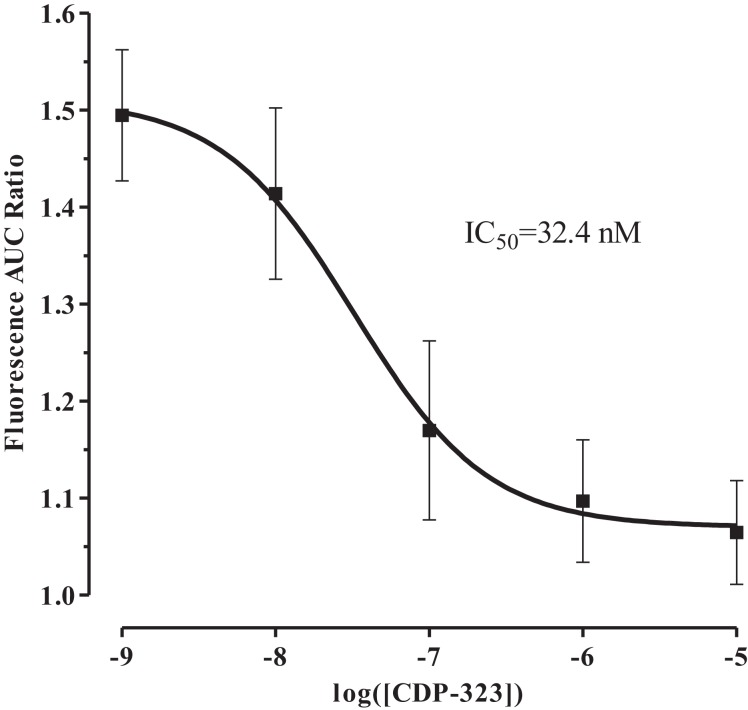
**Effect of CDP-323 on fluid shear stress-mediated calcium flux**. CDP-323 blocks the PICR in a concentration-dependent manner, suggesting that α_4_β_7_/α_4_β_1_ integrin activity plays a direct role in transducing fluid shear stress into a physiological signal in eosinophils. Values represent means ± SEM.

After showing that the PICR is integrin-mediated, we identified the integrin receptor subtype(s) involved in this phenomenon using a novel α_4_β_7_/α_4_β_1_ dual-antagonist known as CDP-323 (or Zaurategrast), which has been shown to be a potent and highly selective blocker of these integrin receptors (35). Calcium flux was measured in eosinophils on fibronectin and pre-treated with CDP-323 (a selective blocker of α_4_β_7_/α_4_β_1_ integrin receptors) and stimulated with fluid perfusion. Consistent with our previous findings, eosinophil pre-treatment with CDP-323 blocked the PICR in a concentration-dependent manner (Figure 5; IC_50_ = 32.4 nM).

### Interaction Between Perfusion-Induced and Agonist-Evoked Calcium Responses

The finding that onset of perfusion alone augments [Ca^2+^]_i_ by mobilizing intracellular calcium stores is functionally interesting in light of the central role of Ca^2+^ as a secondary messenger in the eotaxin-1 signal transduction cascade (36, 37). We therefore sought to investigate whether or not these mechanosensitive integrin receptors engage in cross-talk with the eotaxin-1 signaling pathway and/or whether integrins can affect eotaxin-1-mediated eosinophil activation.

**Figure 6:**
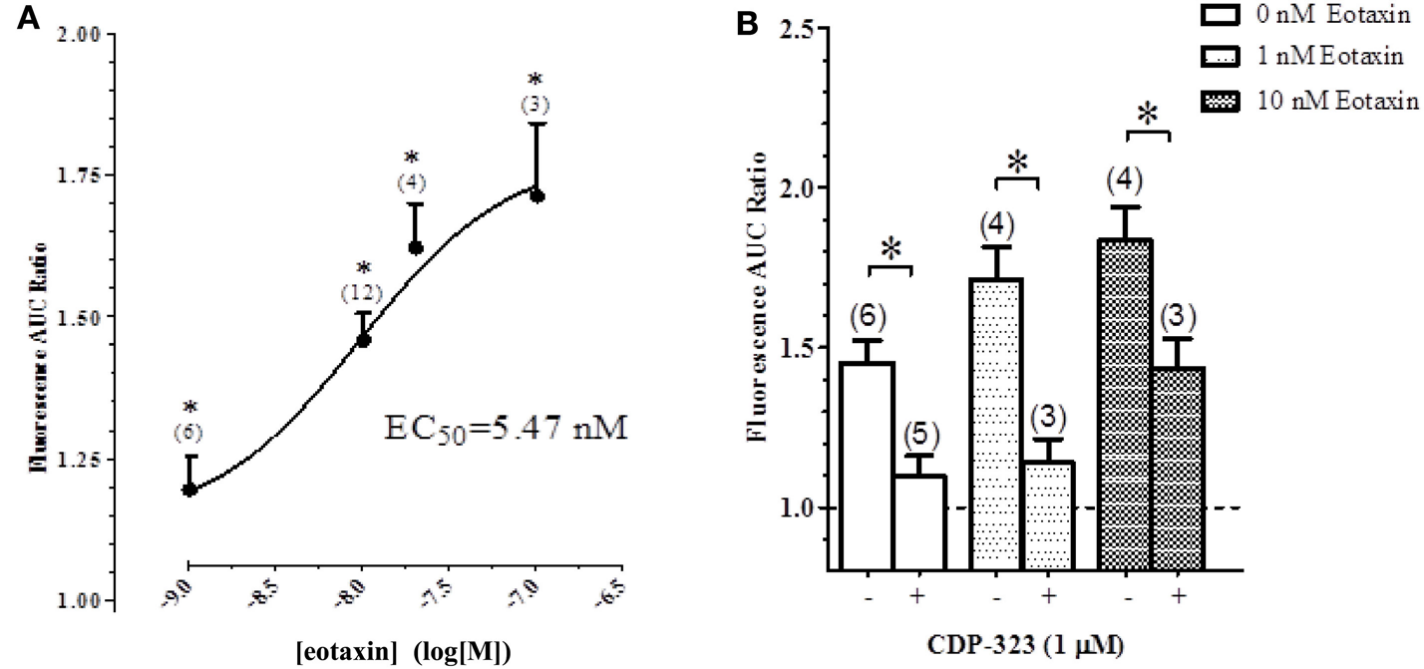
**Effect of CDP-323 on the eotaxin- and perfusion-induced calcium response**. **(A)** Elevation of [Ca^2+^]_i_ in eosinophils on fibronectin (measured as AUC) is augmented by eotaxin in a concentration-dependent manner. **(B)** PICR in the presence of varying concentrations of eotaxin with or without CDP-3223. Sample sizes enumerated in brackets.

To quantify the eotaxin-evoked calcium response, we measured the total AUC in the tracings over a period of 10 min. Application of a physiologically relevant concentration of eotaxin-1 to eosinophils increased the AUC in a concentration-dependent manner (Figure 6A).

In another experiment, cells were pre-treated with CDP-323 before examining eotaxin-evoked fluorimetric responses: CDP-323 significantly inhibited both the PICR and the eotaxin-evoked responses (Figure 6B).

## Discussion

There have been many studies of adhesion and migration of leukocytes on a variety of substrates. These describe the involvement of cell adhesion molecules and various signaling events triggered by shear stress, including elevations of cytosolic levels of calcium. However, there are important gaps in this large body of knowledge. First, the majority of such studies have been done using relatively purified populations of neutrophils, and several groups have since shown that eosinophils can respond in markedly different fashion. Second, many of these studies have examined migration and the calcium response in large populations of cells, which average out the changes seen in individual cells and thereby may occlude important mechanistic insights.

In this study, we examined cell shape, [Ca^2+^], adhesion, and migration in individual human eosinophils.

We first noted that adherent eosinophils responded to a shear stress stimulus (imposed by a sudden onset of flow past the cells) with a transient elevation of [Ca^2+^]. Even though the cells would have experienced the shear stress instantaneously upon onset of flow (as indicated by the immediate sweeping away of debris and other cells in the field of view), this PICR was delayed for several minutes, depending on the rate of flow. The delay may represent the time required for integrin-activation to trigger a signaling cascade that culminates in activation of PLC and production of the calcium-mobilization second messenger IP_3_ (21). This PICR was accompanied by cell flattening and migration.

Our data further permit speculation about the nature of the interactions between shear stress, the PICR, and the morphological changes. The fact that the cells could be adherent prior to flow (i.e., such that they were not swept away with other surrounding cells and debris upon onset of flow) without undergoing shape changes or migration (Figures 1B,C) suggests that binding of cell adhesion molecules alone is not sufficient for cell activation. Furthermore, eosinophils studied prior to flow or in the absence of flow rarely exhibited elevations in [Ca^2+^] (Figure 1A), and did not undergo flattening or migration (Figures 1B–E), suggesting that the flow stimulus itself is essential for triggering these changes. The increase in cell area develops more quickly and is more pronounced in cells adhered to fibronectin (Figure 1C) compared to those on glass (Figure 1B), suggesting involvement of fibronectin. Disruption of fibronectin binding using RGD tripeptides (Figure 4), or of blockade of integrin function (Figure 5), compromised the PICR, and preventing the change in [Ca^2+^] by loading the cells with BAPTA completely prevented the morphological changes following induction of flow (Figure 1E).

Altogether, our data suggest that a mechanotransduction event involving integrins and fibronectin triggers the morphological changes, and that the latter require elevation of [Ca^2+^].

Analysis of unattached eosinophils that suddenly became adherent during video recording provide further insight into the possible functional consequence of the PICR. We noted that all unattached cells which suddenly adhered within the field of view (always followed by the same morphological changes described above) also appeared to be in the middle of a transient elevation of [Ca^2+^] (Figure 2): this further suggests that the PICR was also enhancing cell adhesion. Others have also proposed Ca^2+^ as a regulator of eosinophil adhesion (21, 38). However, those previous studies did not show the close temporal correlation between the calcium-transient in single cells and the subsequent morphological changes, as we have done here. Our observation that the PICR in these cells had a similar delay compared to the fully adherent cells described above argues against the PICR being a result of membrane damage following dislodging of the cells from the glass slide: tearing of the membrane would lead to an immediate flooding of the cytosol with external Ca^2+^ and an immediate fluorescent response, not a delayed one. Instead, we interpret these observations to indicate that the cells were only loosely adherent prior to flow, if at all, and that their adhesivity was increased enough to suddenly arrest their forward movement.

Migration was increased nearly twofold in response to perfusion (Figure 3); the overall direction of that increased motility seemed to be not correlated with the direction of bulk fluid flow. The nearly twofold increase in cell migration that we observed in eosinophils in the presence of fluid perfusion did not exhibit any specific directionality (Figure 1C). In contrast to this, intravital microscopy studies have shown that neutrophils crawl on the luminal surface of the microvasculature perpendicularly to the direction of the fluid flow (39). In a limited set of experiments, we found that neutrophils did not exhibit any migrational response to perfusion (data not shown). Eosinophils and neutrophils also differ with respect to the nature of the change in cell shape in response to shear stress: the former undergo increased cell area and flattening (this study), while the latter decrease in cell area and become more spherical (40). Likewise, eosinophil rolling is mediated by l-selectin and VLA-4, whereas that in neutrophils involves only l-selectin (41). Altogether, then, it is clear that findings made in neutrophils cannot be assumed to apply also to eosinophils: unfortunately, the bulk of the available literature on adhesion and migration, and the signaling events underlying those functions, is dominated by work done using the former cell type.

The fact that fluid perfusion uniquely augments [Ca^2+^]_i_ in eosinophils suggests that the PICR constitutes a *bona fide* physiological mechanism that controls eosinophil function in a way that may be of biological/pathological significance (42, 43). Mechanical stress is known to non-specifically distort the cell membrane and to thereby activate cells by inducing Ca^2+^-fluxes in cardiac myocytes, endothelial cells, and sensory and motor neurons (26, 44–47). Pharmacologically blocking cell surface integrin receptors attenuated the PICR in a concentration-dependent manner, however, indicating that the PICR is a receptor-dependent phenomenon rather than a non-specific consequence of perfusion-induced membrane perturbation. This supports the view that shear stress alone stimulates the down-stream production of second messengers via a mechanical pathway that operates independently of the CCR3 signal transduction cascade and raises the intriguing possibility that eotaxin-1-binding alone may not be necessary in order to activate eosinophils.

The role of integrins as mechanosensors of shear stress is well-acknowledged in the literature (27, 48–51). In particular, some groups have highlighted the role of integrins in mediating the flow-dependent adhesion of eosinophils. Barthel et al. (7) have shown that eosinophils adhere to glass coated with BSA in an α_M_β_2_ integrin-dependent manner. Since α_M_β_2_ integrin expresses the RGD tripeptide binding sequence, part of the RGD-mediated decrease in the PICR may indeed be attributable to blockage of the α_M_β_2_ integrins (28, 30, 31). Previous studies using flow cytometry and monoclonal antibodies, however, indicate that eosinophils most highly express α_4_β_7_/α_4_β_1_ integrins (52, 53). The expression pattern of eosinophil adhesion receptors is similar to that of other leukocytes (52, 54). Unlike neutrophils, however, eosinophil maturation culminates in the expression of functional α_4_β_7_ integrin receptors (55). This unique receptor expression pattern may underlie the selective migration of eosinophils to the airways during allergic inflammatory responses, motivating the need to further investigate the mechanosensitive properties of this family of integrin receptors. The fact that the highly selective α_4_β_7_/α_4_β_1_ integrin antagonist, CDP-323, blocks the PICR ultimately corroborates these experimental findings and suggests that these integrin receptors fulfill a unique physiological role that extends beyond their capacity to bind adhesion molecules on the endothelium or in the ECM. In the presence of fluid perfusion, CDP-323 can dramatically abolish the Ca^2+^-response normally elicited by eotaxin. This observation suggests that the signaling program of eotaxin-1 is influenced by the presence of fluid shear stress and the mechanosensitive α_4_β_1_ and/or α_4_β_7_ integrins.

The shear stress to which eosinophils were exposed in our preparation fundamentally results from the frictional forces generated by the cells’ collision with the glass and/or other cells, in addition to the rate at which the fluid is perfused into the flow chamber (56). In the body, physiologically relevant shear stress might arise as eosinophils encounter altered blood flow or as they collide with the walls of the inflamed microvasculature (56).

Altogether, we interpret these observations within an integrated physiological response, as follows. When the cells are suspended in the circulation, they sense little or no shear stress since they are entirely suspended within the circulation. As the cell travels through the capillary toward the site of inflammation, however, integrins and other cell adhesion molecules on activated endothelial cells begin to slow the eosinophil down, which in turn markedly increases shear stress experienced by the eosinophil. Integrin-activation, and/or some other shear stress sensor, then triggers a PICR, strengthens adhesion/attachment, and induces shape change and migration; furthermore, all of these responses are enhanced by eotaxin. As such, the PICR and signaling ligands can work together and synergize in the process of moving the eosinophils out of the circulation and toward their intended site of action.

In conclusion, we show for the first time that human eosinophils respond to a sudden perceived increase in flow by exhibiting a Ca^2+^-flash and a number of concurrent mechanical changes, including adhesion, cell shape change, and migration. Furthermore, there is cross-talk between these phenomena and eotaxin-induced responses. These findings have great importance in understanding the mechanisms underlying eosinophil extravasation in response to a chemotactic stimulus.

## Author Contributions

MA carried out the majority of the experimental work; MS contributed essential data as well. MA, RS, GG and LJ provided intellectual and editorial guidance.

## Conflict of Interest Statement

All authors declare that the research was conducted in the absence of any commercial or financial relationships that could be construed as a potential conflict of interest.

## Supplementary Material

Figure S1 **An overview of experimental set-up**. **(A)** Cells were placed on a 5 cm × 4.5 cm glass cover slip. Two thin strips of silicone (red arrows) were then applied diagonally, onto which a second 2 cm × 4 cm glass slide was gently placed. The “sandwich” preparation provided a gap through which drug-containing physiological salt solution could be drawn towards the cells by capillary action (yellow arrow). **(B)** Drugs and fluids were applied at one corner and aspirated via suction at the opposite corner. The rate at which drugs/fluids perfused through the preparation was controlled by adjusting the heights of the reservoirs relative to the preparation (not shown). Fluid that was drawn through the chamber was siphoned away by vacuum suction placed at the opposite corner.

Video S1 **Video recording showing calcium flash and shape change in response to flow**. Upper panel: fluorimetric recording showing cytosolic [Ca^2+^]. Lower panel: standard transmitted image showing the cell shape. Note: real-time length of experiment is 10 min; playback of recording is greatly accelerated. Cells are rounded and immobile during the first 5 min of the experiment (condensed into the first 6 s of this video). Onset of flow begins at *t* = 6 s, and is indicated by the movement of non-adherent cells from left to right. This is followed by a calcium flash approximately 1 min later, and then cell flattening (with consequent increase in cell area) and migration.
