# Supplementary material for: Integrins are Mechanosensors That Modulate Human Eosinophil Activation
Source: Front Immunol. 2015 Oct 20;6:525. doi: 10.3389/fimmu.2015.00525 (PMC4611147; doi:10.3389/fimmu.2015.00525)
Supplement: Figure S1 — An overview of experimental set-up. (A) Cells were placed on a 5 cm × 4.5 cm glass cover slip. Two thin strips of silicone (red arrows) were then applied diagonally, onto which a second 2 cm × 4 cm glass slide was gently placed. The “sandwich” preparation provided a gap through which drug-containing physiological salt solution could be drawn towards the cells by capillary action (yellow arrow). (B) Drugs and fluids were applied at one corner and aspirated via suction at the opposite corner. The rate at which drugs/fluids perfused through the preparation was controlled by adjusting the heights of the reservoirs relative to the preparation (not shown). Fluid that was drawn through the chamber was siphoned away by vacuum suction placed at the opposite corner. [file image_1.pdf]

(A) (B)

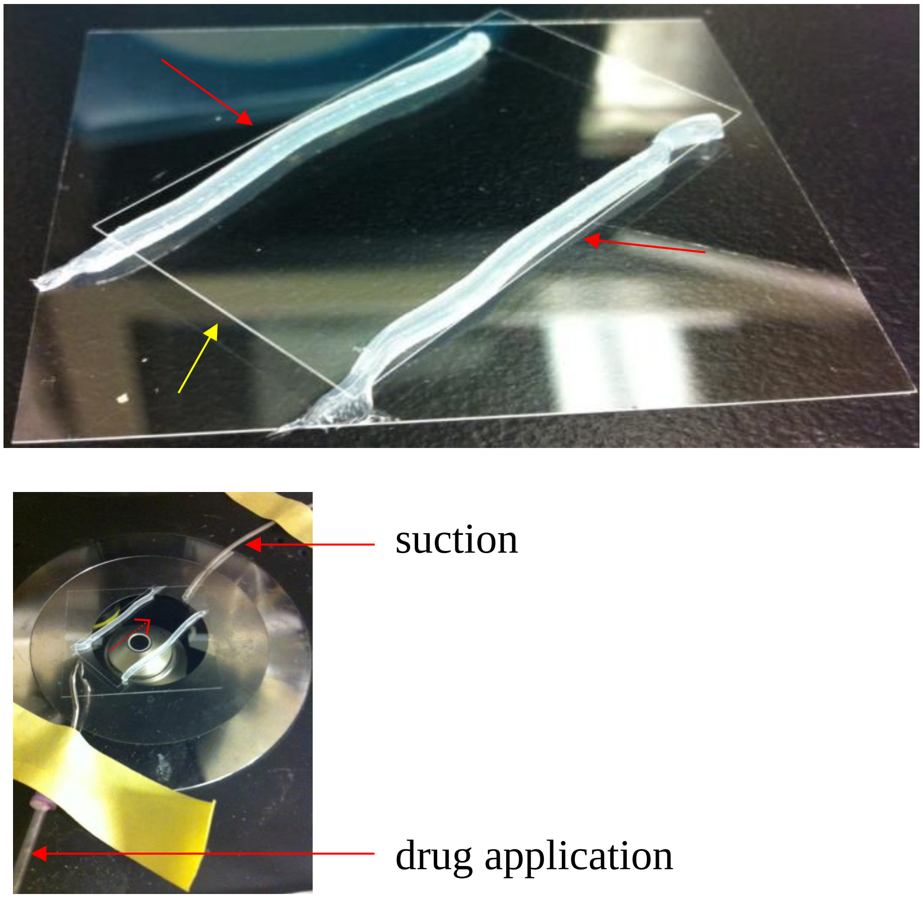

<u>Supplementary Figure 1</u>. Overview of experimental set-up. (**A)** Cells were placed on a 5 x 4.5 cm glass cover slip. Two thin strips of silicone (red arrows) were then applied diagonally, onto which a second 2 x 4 cm glass slide was gently placed. The “sandwich” preparation provided a gap through which drug-containing, physiological salt solution could be drawn towards the cells by capillary action (yellow arrow). **B**: Drugs and fluids were applied at one corner and aspirated via suction at the opposite corner. The rate at which drugs/fluids perfused through the preparation was controlled by adjusting the heights of the reservoirs relative to the preparation (not shown). Fluid that was drawn through the chamber was siphoned away by vacuum suction placed at the opposite corner.
